# Ways of Viewing Pictorial Plasticity

**DOI:** 10.1177/2041669517699220

**Published:** 2017-04-06

**Authors:** Maarten W. A. Wijntjes

**Affiliations:** Perceptual Intelligence Lab, Industrial Design Engineering, Delft University of Technology, The Netherlands

**Keywords:** stereopsis, three-dimensional vision, synopter, depth perception, pictorial space, plastic effect

## Abstract

The *plastic effect* is historically used to denote various forms of stereopsis. The vivid impression of depth often associated with *binocular* stereopsis can also be achieved in other ways, for example, using a synopter. Accounts of this go back over a hundred years. These ways of viewing all aim to diminish sensorial evidence that the picture is physically flat. Although various viewing modes have been proposed in the literature, their effects have never been compared. In the current study, we compared three viewing modes: monocular blur, synoptic viewing, and free viewing (using a placebo synopter). By designing a physical embodiment that was indistinguishable for the three experimental conditions, we kept observers naïve with respect to the differences between them; 197 observers participated in an experiment where the three viewing modes were compared by performing a rating task. Results indicate that synoptic viewing causes the largest plastic effect. Monocular blur scores lower than synoptic viewing but is still rated significantly higher than the baseline conditions. The results strengthen the idea that synoptic viewing is not due to a placebo effect. Furthermore, monocular blur has been verified for the first time as a way of experiencing the plastic effect, although the effect is smaller than synoptic viewing. We discuss the results with respect to the theoretical basis for the plastic effect. We show that current theories are not described with sufficient details to explain the differences we found.

## Introduction

The term stereopsis is often associated with the perception of depth based on binocular disparities. However, it has recently been argued (Vishwanath & Hibbard, 2013) that the subjective impression evoked by stereo pictures very much resembles the depth impression of monocular aperture viewing. This *paradoxical* (Claparède, 1904; Koenderink, van Doorn, & Kappers, 1994) stereoscopic impression had been predicted before (Ames, 1925; Ebbinghaus, 1902) but was never directly and qualitatively compared with stereoscopic picture viewing. Monocular aperture viewing is a single case of a collection of viewing modes that achieve this effect, also known as the *plastic* effect (Higashiyama & Shimono, 2012; Koenderink, Wijntjes, & van Doorn, 2013). These ways of viewing include the following:

1. Monocular viewing (even better when using an aperture).
2. Optically decrease interocular distance (Javals’ iconoscope).
3. Increase the viewing distance.
4. Looking through an artificial pupil.
5. Change accommodation to infinity (using a lens).
6. Change convergence to parallel.
7. Introduce blur in one eye.
8. Looking through a mirror.
9. Looking through a synopter.

These ways of viewing have all been discussed earlier (Ames, 1925; Blundell, 2015; Schlosberg, 1941), including a concise overview of our own (Wijntjes, Füzy, Verheij, Deetman, & Pont, 2016).

What all these viewing modes have in common is that they change or eliminate the nonpictorial depth cues of accommodation, vergence, and binocular disparity. When viewing a picture normally, these cues will signal that the stimulus is flat. Although pictures are indeed physically flat, they often contain pictorial depth cues that create an impression of three-dimensional space, also known as pictorial space. In these cases (that occur continuously in our daily lives), the visual system is confronted with a cue conflict situation: Nonpictorial cues signal a *flat* stimulus, while pictorial cues signal a *deep* stimulus. A common explanation of the plastic effect is that perceived depth is the result of (possibly weighted) averaging of physical flatness and pictorial depth (Ames, 1925). Thus, removing the physical flatness would result in a relatively increased impression of depth, which is the plastic effect. This explanation predicts quantitative changes in depth as has been found by Koenderink et al., (1994) but could not be verified by Vishwanath & Hibbard, (2013). An alternative explanation of the plastic effect is the “absolute depth scaling hypothesis” (Vishwanath, 2014) which does not predict a quantitative depth change. According to this hypothesis, perturbing the reliability of depth cues specifying the flat picture surface results in ascribing distance information deriving from absolute depth cues (like accommodation, vergence, and vertical disparities) to pictorial depth cues allowing them to be scaled in a manner similar to the scaling of binocular disparities. The similar scaling mechanism is according to Vishwanath (2014) responsible for the phenomenological similarity.

Not all of the viewing modes have been empirically investigated, and some of these (although mentioned separately in the literature) are largely similar. For example, Javals’ iconoscope uses four mirrors to decrease the virtual interocular distance, while the synopter completely nullifies the virtual interocular distance due to a half translucent mirror. The viewing modes that have attracted the least amount of attention in the literature seem to be the artificial pupil (4) and monocular blurring (7). The concept of the artificial pupil originates from Ames (1925) who recommends an aperture size of 2 mm or larger. Similar to photography, the sharpness of the retinal image depends less on the accommodation for small aperture sizes than for large apertures. The limiting case is the pinhole camera where no lens is needed. Using a small artificial pupil will thus decrease the reliability of the accommodation cue. Ames (1925) notes that the eye seems to automatically change accommodation (because it cannot find a sharp image) resulting in apparent changes of object size. Vishwanath and Hibbard (2013) used a much larger aperture (12–15 mm) keeping potential depth information from accommodation reliable. This raises the question of whether reducing the reliability even more would substantially improve the plastic effect. Aside from these theoretical considerations, we informally found that looking through a very tiny aperture is rather cumbersome. The reader can easily verify this him or herself by punching holes in an opaque sheet of cardboard (we used a hole puncher that ships with a DIY music box kit).

The other *neglected* viewing mode appears more promising: monocular blurring. Here, one eye receives a blurred image while the other eye receives a sharp image. This binocular sharpness difference disturbs the stereo correspondence problem, that is, the accurate matching of retinal image features from which disparities are computed. The obvious apparent downside is that the observer may become aware of the binocular conflict and experience alternating rivalry between the sharp and blurred image. However, it is well known (Levelt, 1966) that in this specific case of a sharp and blurred image, the sharpest image generally dominates awareness. Therefore, the observer should theoretically not be too much bothered by an alternating percept.

In the study presented here, we wanted to test this aforementioned viewing mode and verify whether the plastic effect can be induced by monocular blur. Initially, we planned to use the synopter as a baseline condition for the plastic effect. The synopter is an optical device invented by von Rohr (1907) that gives both eyes a similar viewpoint and thus eliminate disparity information. Furthermore, it forces the eyes to have parallel binocular vergence. Previously, we investigated the contribution of pictorial cues to the plastic effect by using a variety of paintings using a newly designed synopter (Wijntjes et al., 2016).

While the synopter appears to be a good candidate to compare new, untested viewing modes with, we realized that the synopter itself has never been thoroughly tested against a baseline condition of normal binocular picture viewing. In our previous study (Wijntjes et al., 2016), we let observers compare synoptic viewing with free viewing. Although we found coherent results among observers for a large variety of paintings, we did not address the potential contribution of a placebo effect: Maybe merely viewing through a box and thereby introducing an artificial frame was evoking the sensation of stereoscopic depth, and not the optical design itself. Thus, for a fair comparison between the synopter and monocular blur we should also involve normal binocular viewing.

It is not straightforward to make a prediction which viewing mode will result in the largest plastic effect. The reason is that monocular blur and synoptic viewing perturb nonpictorial depth cues in different ways. Monocular blur theoretically alters the *sensitivity* of both vergence and disparity signals, while synoptic viewing introduces a *bias* in the vergence and disparity signals. Theories explaining the plastic effect (e.g., Ames [1925] or Vishwanath [2014]) do not make a distinction between these two ways of altering the nonpictorial signals. Therefore, our experiment will not settle the debate which theory best describes the plastic effect, neither are we able to predict which viewing mode may results in the strongest plastic effect. However, the current study will provide empirical insights that may help theory formation concerning the plastic effect. Furthermore, the study will also reveal whether it is more efficient to alter the bias (synopter) or sensitivity (monocular blur) to achieve the optimal plastic effect.

## Methods

### Venue and Context

The experiment took place during a three-day festival called Campingflight to Lowlands, in 2015. About 48,000 people visited this festival, which is primarily based on pop music performances but also various other kinds of cultural activities. Our experiment was part of the Lowlands Science program and was located in the *ArtTube* tent, a venue where visitors could take part in various art-related activities and experiments. The experiment was conducted during 3 consecutive days at daytime between 12.00 and 18.00.

### Participants

A total of 193 visitors participated in our experiment; 97 were male, 93 were female, and 3 did not specify their gender. Average age was 30 years with the youngest participant being 15 and the oldest being 63. Participants were asked whether they had used something “that could influence their judgments,” 54 responded affirmative. Specifying their usage, 46 responded that they had used alcohol (13 consumed one unit, 2 consumed two units, 1 consumed three units, and 30 did not specify the total). A total of six had used cannabis (four in combination with alcohol). Binocular stereo vision was assessed through self-report: Five responded to have ill binocular stereo vision.

### Apparatus and Stimuli

**Figure 1.**
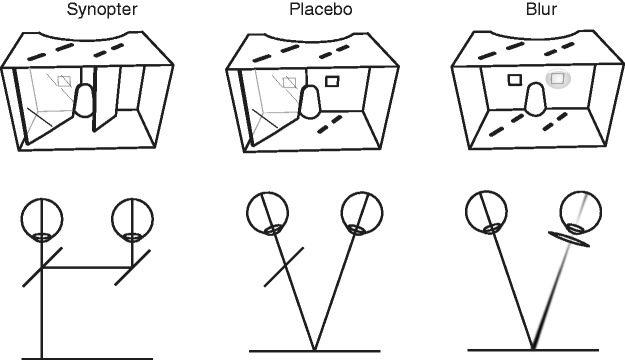
The three devices used in the experiment. Top row depicts them from the point of view of the stimulus (the observers’ face should be behind the device). Bottom row shows the qualitative optics involved.

We used three types of viewing devices. From the outside, all three looked similar, but the interior was very different. The first device (see Figure 1) was a synopter close to described by Wijntjes et al. (2016). We used a wooden (plywood) embodiment with a black spray painted interior. The second device was similar to the synopter but we removed the full mirror in the left eye. Therefore, the right eye was looking through a half mirror while the left eye had no obstruction. With this design, stereovision is not impaired and should work normally: It is a placebo synopter. We kept the half mirror on the right eye to introduce luminance differences between the two eyes, which also occurs in our normal synopter due to the low beam splitting quality of the acrylate half mirror. The third device had a similar embodiment as the other two devices but did not have any mirrors. Instead, we glued a reading glass lens of +3 diopter behind the right eye aperture. This lens introduces blur when viewing at the distance we used in our experiment (approximately 2 m).

**Figure 2.**
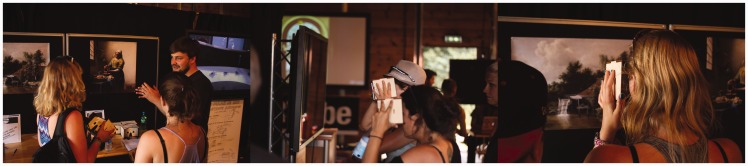
The experimental scene. On the left, an experimenter explains the experiment with the stimuli visible in the background. Middle and right pictures show observer holding the devices to look at either or the stimuli.

As stimuli, we used matte prints of two paintings: The Watermill (c. 1664) by Meinder Hobbema and The Milkmaid (c. 1660) by Johannes Vermeer, both paintings are in the collection of the Rijksmuseum in Amsterdam. They were printed on A2 sized paper, resulting in a size of 59.4 cm wide and 42 cm high for the Watermill, and 42 cm wide and 47.3 cm high for the Milkmaid. The prints were attached to poster boards at approximately eye height (about 170 cm). Pictures documenting the experimental environment are shown in Figure 2.

### Procedure

Observers were first explained that we were investigating various devices that had been hypothesized to increase pictorial depth. We asked the observers to assess the three devices by rating them between 1 (no effect) and 10 (huge depth effect). They were informed that the absolute values of the ratings were not very important to us, and that we were primarily interested in the relative differences between the values. To assess the effect of the viewing devices, the observers were asked to compare perception with and without device by alternately looking through the device, and looking past it.

Observers were instructed to look at approximately 2 m distance, although in practice this distance could vary somewhat. The devices were presented on a table, and the observers could freely alternate between them. The order of viewing was random in the sense that the devices were randomly presented on the table; the observers chose their own order. Most observers were either looking at the Watermill or the Milkmaid. Only at Day 2 did we start logging this independent variable, which amounts to about 100 cases.

The experiment was performed in agreement with the Declaration of Helsinki and approved by the TU Delft Human Research Ethics Committee.

### Data Analysis

Responses were analyzed using a repeated measures analysis of variance with age as covariate, and gender, stimulus, and reported drug use as between-visitor factors.

## Results

**Figure 3.**
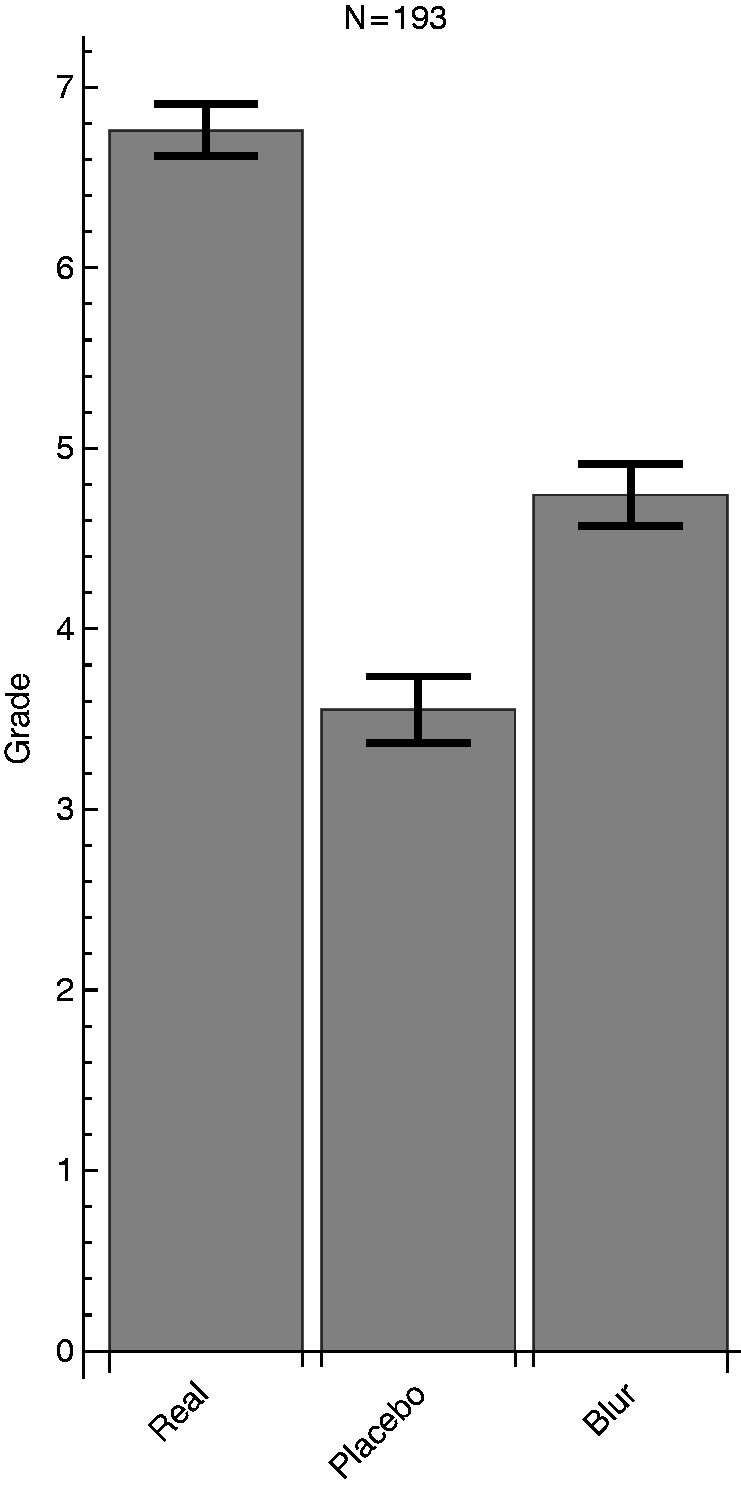
Average ratings and standard errors for the three viewing conditions.

The average ratings per viewing device are displayed in Figure 3. As can be seen, the real synopter was rated highest, the placebo version the lowest and the blur device was rated in between. A statistical main effect was confirmed by the (Greenhouse-Geisser corrected) analysis of variance: *F*(1.921, 312)=11.831, *p* < .05. Furthermore, Bonferroni-corrected pairwise comparisons revealed that all conditions were significantly different from each other (*p* < .05). Neither the covariates age, nor the between-subject factors gender, stimulus and reported drug use had a significant influence on the ratings.

**Figure 4.**
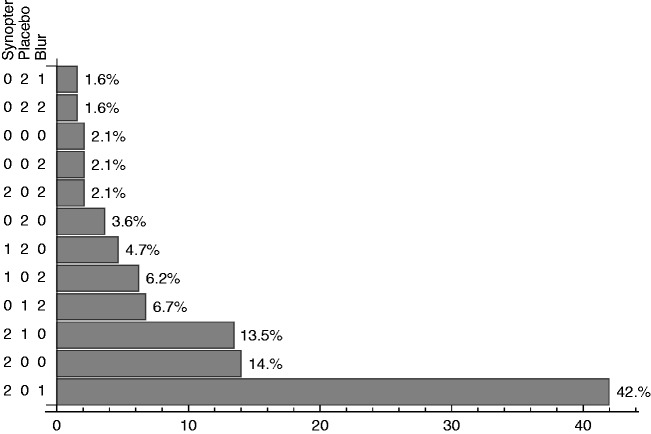
Histogram of the various orderings that were present in the results. Highest is denoted by 2, middle by 1, and lowest by 0. If a number is presented twice, these viewing modes were graded similarly. As can be seen, synopter highest (2), monocular blur middle (1), and placebo lowest (0) dominate the data.

Because we had instructed the participants to primarily focus on the relative differences between their ratings, and not so much on the absolute values, we also (descriptively) analyzed the subjective ordering. In Figure 4, we plotted a histogram of the various ordering combinations. The numbers on the *y* axis denote the order: 2 is highest, 1 intermediate, and 0 lowest. As can be seen, the synopter scored highest in almost 70% of the cases (the sum of the lowest three bars). Furthermore, the configuration with the synopter highest, placebo lowest, and monocular blur intermediate (lowest bar, 42%) is by far occurring most often: It is three times more than the follow-up configuration (synopter highest, no difference between placebo and monocular blur: 14%).

## Discussion

We found that both monocular blur and synoptic viewing induced the plastic effect in comparison with the baseline condition of normal binocular viewing. Furthermore, the synopter evoked a larger plastic effect than monocular blur. Therefore, we can conclude that previous findings (e.g., Wijntjes et al., 2016) cannot be attributed to a placebo effect and that the hitherto neglected** viewing mode of monocular blur indeed enhances the impression of pictorial depth as predicted (Ames, 1925; Schlosberg, 1941).

Based on our (informal) experience with synopter demonstration events, the large difference between the synopter and the placebo hardly comes as a surprise. Often, observers express surprise when looking at a painting through a synopter. However, it had never been experimentally verified whether these expressions could be attributed to some kind of placebo effect. Since our placebo version has a similar embodiment as the synopter, we can also safely argue that the synoptic effect cannot be attributed to looking through a box that masks much of the periphery. It may certainly help with concentrating on an artwork while wearing *blinkers* (used to focus the horse gaze), but it is not solely responsible for the plastic effect.

We also found that monocular blur increases the plastic effect, in comparison with the baseline condition of the placebo. Perturbing binocular vergence and disparities through blurring one of the images increased the impression of pictorial depth. This finding implies the design of a very simple plastic effect device: A pair of reading glasses and with one of the lenses removed. However, we found that the effect of monocular blur is substantially weaker than the synopter. It may also be noteworthy that we never observed expressions of spontaneous surprise associated with synoptic viewing. This may be reflected by the *absolute* rating values. Although observers were instructed to primarily focus on the *relative* grades, it is likely that observers were inclined to use an absolute scale. Since the scale used on the experiment was between 1 and 10, it is probable that observers referred to the examination grades used in the Netherlands, that fall between 1 and 10. In this system, 5 and lower is insufficient, 6 and higher is sufficient. Taking this into account, monocular blur scores *insufficient*, while the synopter passes the exam.

During the experiment, we picked up introspective comments indicating unforeseen negative side effects of the synoptic parallel vergence. Sometimes, observers responded that they saw two images when looking through the synopter. This occurs when observers set their vergence at the physical viewing distance. Possibly, accommodation or familiar size (the images had been seen when entering the space and may have a *typical* size of an average painting or poster) causes this vergence setting to occur. We heard from at least one participant that he rated the placebo higher because it did not give him double images. As can be inferred from Figure 3, there are four types of orderings in which the placebo was higher rated than the synopter. These four amount to 11.4% of the observers. Although this is not a large amount, it is important to consider that these cases may mitigate an actually larger difference between the synopter and placebo condition.

**Figure 5.**
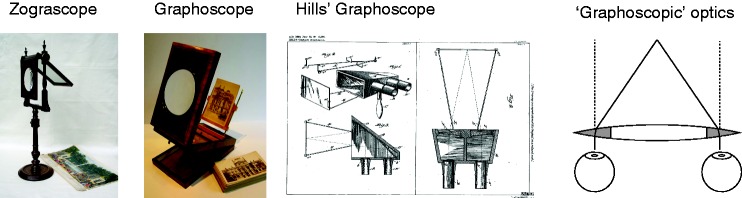
Illustration of the zograscope and graphoscope, including Hills’ variation. The principle relies at least partly on the prismatic nature of the lens edges (Michaud, 1908) illustrated at the right.

Although accounts referring to the plastic effect have existed in the literature for over a hundred years, it has hardly been studied. The most salient experimental findings are changes in pictorial relief using either a synopter (Koenderink et al., 1994) or a zograscope or graphoscope (Koenderink et al., 2013) and the experiential similarity between binocular stereo and monocular aperture viewing (Vishwanath & Hibbard, 2013). The aforementioned viewing devices are illustrated in Figure 5. Besides the empirical findings, there is relatively little theoretical work. As outlined in the introduction, the two main theories explaining the plastic effect (cue combination and absolute depth scaling) do not seem to have been described in sufficient detail to quantitatively predict effect differences. One difficulty is the distinction between sensitivity and bias of the physiologically altered cues. For monocular blur, the sensitivities of the vergence and disparity cues are decreased. For synoptic viewing, the vergence and disparity cues are biased. In the latter case, the biasing of the cues toward infinity may result in a decrease of their reliability. Therefore, in both viewing modes, the reliability of physiological depth cues is decreased but in very different ways. This made it difficult to compare quantitatively in this study. Nevertheless, it is not impossible to design future experiments that address this by systematically investigate nonpictorial cues. For example, sensitivity studies can be conducted on the various ways of viewing to quantify the size of variances introduced by the viewing modes. Also, it is possible to alter the vergence of the synopter toward the viewing distance without affecting the disparities, which could reveal the contribution of binocular vergence. Another interesting device that may help answering these questions is Hill’s (1898) graphoscope which forces vergence to be parallel using prisms, without affecting disparities as shown in Figure 5.

Besides the fundamentally interesting underlying mechanisms of the plastic effect, studies on various ways of viewing the plastic effect also have practical relevance. The consumer interest in three-dimensional displays and virtual reality indicates that people are interested in seeing pictures differently. However, these novel techniques are only applicable when a stereo image pair is available. There are numerous examples of occasions where this is impossible. Our personal favorite application is looking at paintings in a museum, but also more mundane activities such as viewing personal photos on a screen may become more interesting when the plastic effect is present. Thus, studying and applying old techniques and inventions concerning the plastic effect may be a valuable addition next to the high-tech advances of virtual reality, as has recently been argued by Blundell (2015). The current findings in combination with our previous study (Wijntjes et al., 2016) indicates that the synopter version we used in these studies is a good candidate for viewing pictures at distances of about 1.5 to 2 m. For presentations on small displays that are close to the eyes, other devices may be useful, such as the Verant lens (also by von Rohr), or Hill’s graphoscope. And possibly there is some space left for novel devices yet to be invented.
